# Decoding a Million Genomes: Unveiling the Protein-coding Landscape and Its Implications for Precision Medicine

**DOI:** 10.2174/0113892029356185241216063635

**Published:** 2025-01-08

**Authors:** Jinwei Zhang

**Affiliations:** 1 Institute of Biomedical and Clinical Sciences, Medical School, Faculty of Health and Life Sciences, University of Exeter, Hatherly Laboratories, Streatham Campus, Exeter, EX4 4PS, UK;; 2 State Key Laboratory of Chemical Biology, Research Center of Chemical Kinomics, Shanghai Institute of Organic Chemistry, Chinese Academy of Sciences, 345 Lingling Road, Shanghai, 200032, China

**Keywords:** Protein-coding variation, rare genetic variants, precision medicine, exome sequencing, population genetics

## Abstract

The study by Sun *et al.* , which sequenced exomes from 983,578 individuals, provides a comprehensive resource on protein-coding genetic variation. This commentary examines the key findings, including rare biallelic variants and loss-of-function intolerant genes, while emphasizing their implications for gene splicing, human knockouts, and disease-associated genes. Additionally, we discuss how these insights propel advancements in precision medicine and suggest future research directions, particularly in the study of non-coding DNA and regulatory RNAs at population scales.

## 1 INTRODUCTION

The human genome is a reservoir of genetic information shaped by millions of years of evolution. High-throughput sequencing technologies have revolutionized the ability to catalog and interpret genetic variation, particularly in the context of health and disease [1]. The study by Sun *et al.* represents a significant leap forward, analyzing exomes from 983,578 individuals to uncover a detailed landscape of predicted loss-of-function (pLOF) variants [2]. By identifying rare variants across diverse populations, this dataset establishes a crucial foundation for future research and clinical applications in precision medicine.

## 2 MAIN FINDINGS

**Fig. (1):**
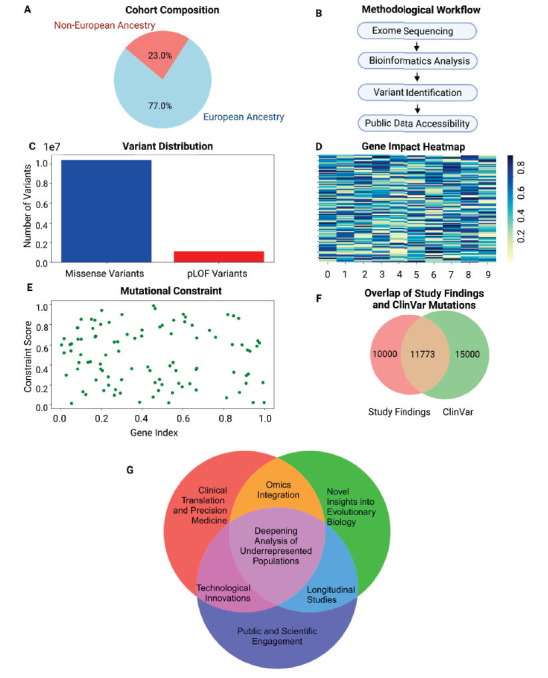
Overview of study design and key findings in protein-coding variation analysis of 983,578 individuals. (**A**) A pie chart showing the demographic breakdown of the 983,578 individuals, highlighting the 23% representation from non-European ancestries. (**B**) A flowchart illustrating the steps from exome sequencing, bioinformatics analysis, and variant identification to public data accessibility. (**C**) A bar graph comparing the number of identified missense and predicted loss-of-function (pLOF) variants. (**D**) A heatmap showing the distribution of rare biallelic pLOF variants across the 4,848 genes, with an emphasis on the 1,751 newly reported genes. (**E**) A scatter plot highlighting the 3,988 loss-of-function intolerant genes and regions of missense depletion in 1,482 genes. (**F**) A Venn diagram illustrating the overlap between the study’s findings and the ClinVar database, highlighting the 11,773 mutations of unknown significance predicted to be deleterious. A graphical representation showing the 3% of individuals with clinically relevant genetic mutations and the potential impact on precision medicine. (**G**) Future perspectives on genomic research and precision medicine. This figure was generated through Biorender.com, data analysis using Python with the available data from Sun *et al*. [2].

Sun *et al.* used exome sequencing to analyze genetic data from a large and diverse cohort, including 23% from non-European ancestries (Fig. **1A**). The dataset, accessible through the RGC Million Exome Browser (https://rgc-research.regeneron.com/me/home), offers comprehensive variant interpretation and frequency analysis (Fig. **1B**). Key findings include the identification of over 10.4 million missense variants and 1.1 million predicted loss-of-function (pLOF) mutations (Fig. **1C-D**) [2], revealing a wealth of previously unexplored genetic variation. Rare biallelic pLOF variants were detected in 4,848 genes, with 1,751 of these being newly reported. Additionally, 3,988 genes were found to be highly intolerant to loss-of-function mutations, challenging prior assumptions about gene tolerance. Regions of missense depletion, identified in 1,482 genes (Fig. **1E**), highlight areas where even single amino acid changes are highly deleterious. The study also sheds light on 11,773 cryptic splice sites previously categorized as variants of unknown significance in the ClinVar database (Fig. **1F**), emphasizing their potential to disrupt gene splicing. These findings underscore the role of mutational constraint in shaping our genetic landscape and demonstrate how such data can guide precision medicine [1, 3].

## 3 PROTEIN-CODING VARIATION AND PRECISION MEDICINE

### 3.1 Implications for Precision Medicine

The findings from Sun *et al.*’s study offer valuable insights for advancing precision medicine. The dataset not only identifies clinically actionable genetic variants in approximately 3% of individuals but also highlights the significant potential for improving diagnostic and therapeutic strategies. By uncovering the prevalence and impact of these variants, the study provides a robust framework for integrating genetic data into clinical practice.

### 3.2 Systems-level Understanding of Disease

This research enhances the systems-level understanding of human disease by exploring the functional impact of genetic variation. The analysis of 4,848 genes with rare biallelic pLOF variants provides natural models for studying gene function and its implications in health and disease. Insights into gene splicing mechanisms are strengthened by the identification of cryptic splice sites, enhancing the precision of genetic testing and variant interpretation. Furthermore, the discovery of loss-of-function intolerant genes offers a foundation for prioritizing potential therapeutic targets, enabling the development of interventions that minimize off-target effects.

### 3.3 Expanding to Non-coding Regions

Beyond protein-coding regions, future efforts should focus on integrating non-coding DNA and regulatory RNAs into this dataset. Such research could uncover additional layers of genetic regulation and provide critical insights into the etiology of diseases influenced by non-coding variants. Combining this exome-focused dataset with transcriptomics and epigenomics can reveal the interplay between coding and regulatory elements, advancing our understanding of complex disorders.

### 3.4 Applications in Clinical Practice

The dataset has broad applications in clinical practice, from enabling more accurate genetic counseling by providing detailed allele frequency data across diverse populations to identifying new drug targets through the overrepresentation of pLOF variants in metabolic pathways. Improved annotations of cryptic splice variants and rare pLOFs further enhance the precision of genetic diagnostics, particularly for conditions that were previously unexplained.

## 4 COMPARISONS WITH RELEVANT STUDIES

Sun *et al.*’s study builds upon previous work, such as the ExAC and gnomAD projects, which provided foundational knowledge on genetic variation in smaller cohorts [3, 4]. Compared to the ExAC study that analyzed 60,706 individuals and the gnomAD project with 141,456 participants, the present study’s scope is significantly larger, encompassing nearly a million individuals. This expansion allows for more accurate estimates of allele frequencies, particularly for rare variants, and offers greater statistical power to detect significant associations. The research also aligns with findings from the UK Biobank and TOPMed programs [5-7], which emphasized the importance of diverse genetic datasets. The inclusion of non-European ancestries in Sun *et al.*’s study is a crucial advancement, providing insights into genetic variation across different populations and enhancing the relevance of findings to global health.

## 5 FUTURE PERSPECTIVES

The dataset generated by Sun *et al.*’s study provides an unparalleled foundation for advancing precision medicine and understanding genetic variation. Integrating this resource with multi-omics data, including transcriptomics, proteomics, and metabolomics, should be a top priority for future study in order to identify the biological processes that connect genetic variations to health and illness (Fig. **1G**). Technological innovations in sequencing and bioinformatics are essential for improving variant detection and functional validation, particularly through tools like CRISPR. Detailed analyses of underrepresented populations will address disparities and reveal population-specific variants. Longitudinal studies are crucial for assessing the clinical relevance of variants over time, while effective clinical translation requires harmonized data workflows and robust computational models. Additionally, exploring non-coding DNA and regulatory RNAs on a population scale will provide new insights into gene regulation and disease processes. Expanding international collaborations and engaging the broader scientific community will accelerate the realization of precision medicine and improve global health equity.

## CONCLUSION

Sun *et al.* ’s work marks a pivotal advancement in human genomics, providing an extensive catalog of protein-coding variation that will inform future research and improve the clinical management of genetic diseases. This resource underscores the importance of continued efforts to bridge the gap between research and clinical practice, ensuring that precision medicine delivers equitable and tangible benefits across all populations.

## AUTHORS’ CONTRIBUTIONS

JZ wrote the initial version of the manuscript, revised and edited the manuscript. The author(s) read and approved the final manuscript.

## ABBREVIATION

pLOF: Predicted Loss-of-function
